# *catena*-Poly[[cadmium-bis­(μ-triethyl­ene­tetra­mine-κ^4^ *N*,*N*′:*N*′′,*N*′′′)-cadmium-(μ-triethyl­ene­tetra­mine-κ^4^ *N*,*N*′:*N*′′,*N*′′′)] hexa­fluoridogermanate]

**DOI:** 10.1107/S1600536811033381

**Published:** 2011-08-27

**Authors:** Guo-Ming Wang, Pei Wang

**Affiliations:** a Teachers College, College of Chemistry, Chemical Engineering and Environment of Qingdao University, Shandong 266071, People’s Republic of China; b College of Chemistry, Chemical Engineering and Environment of Qingdao University, Shandong 266071, People’s Republic of China

## Abstract

The title fluoridogermanate, {[Cd_2_(C_6_H_18_N_4_)_3_][GeF_6_]}_*n*_, was synthesized hydro­thermally. The crystal structure comprises undulated cationic [Cd_2_(TETA)_3_]^4+^ chains (TETA is triethyl­ene­tetra­mine) propagating parallel to [101]. The central Cd^II^ atom is six-coordinated in a CdN_6_ set by three TETA ligands. The isolated [GeF_6_]^2−^ units, serving as counter-anions, occupy the inter-chain spaces and simultaneously link adjacent chains into a three-dimensional network through extensive N—H⋯F hydrogen-bonding inter­actions. One of the ethyl­ene bridges of one TETA ligand is disordered around a twofold rotation axis.

## Related literature

For background to the structures and applications of microporous materials, see: Cheetham *et al.* (1999 ▶); Liang *et al.* (2006 ▶); Su *et al.* (2009 ▶); Zheng *et al.* (2003 ▶); Zou *et al.* (2005 ▶). For previously reported structures containing fluoridogermanate anions, see: Hoard & Vincent (1939 ▶); Brauer *et al.* (1980 ▶, 1986 ▶); Lukevics *et al.* (1997 ▶); Zhang *et al.* (2003 ▶); Wang *et al.* (2004 ▶). For polyamine Cd^II^ coordination complexes, see: Bartoszak-Adamska *et al.* (2002 ▶); Ma *et al.* (2005 ▶); Bose *et al.* (2006 ▶).
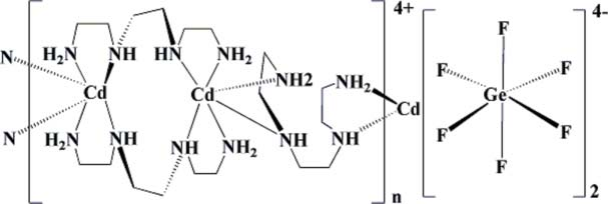


## Experimental

### 

#### Crystal data

[Cd_2_(C_6_H_18_N_4_)_3_][GeF_6_]
*M* *_r_* = 1036.71
Monoclinic, 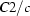

*a* = 16.5034 (3) Å
*b* = 9.1072 (3) Å
*c* = 22.1920 (4) Å
β = 100.354 (5)°
*V* = 3281.14 (14) Å^3^
*Z* = 4
Mo *K*α radiation
μ = 3.20 mm^−1^
*T* = 295 K
0.10 × 0.06 × 0.05 mm

#### Data collection

Bruker APEX area-detector diffractometer
Absorption correction: multi-scan (*SADABS*; Sheldrick, 1996 ▶) *T*_min_ = 0.741, *T*_max_ = 0.857
11087 measured reflections
3385 independent reflections
2642 reflections with *I* > 2σ(*I*)
*R*_int_ = 0.045

#### Refinement

*R*[*F* ^2^ > 2σ(*F* ^2^)] = 0.035
*wR*(*F* ^2^) = 0.076
*S* = 1.00
3385 reflections
218 parameters
H-atom parameters constrained
Δρ_max_ = 0.69 e Å^−3^
Δρ_min_ = −0.55 e Å^−3^

### 

Data collection: *SMART* (Bruker, 1999 ▶); cell refinement: *SAINT* (Bruker, 1999 ▶); data reduction: *SAINT*; program(s) used to solve structure: *SHELXS97* (Sheldrick, 2008 ▶); program(s) used to refine structure: *SHELXL97* (Sheldrick, 2008 ▶); molecular graphics: *DIAMOND* (Brandenburg, 2001 ▶); software used to prepare material for publication: *SHELXTL* (Sheldrick, 2008 ▶).

## Supplementary Material

Crystal structure: contains datablock(s) global, I. DOI: 10.1107/S1600536811033381/wm2519sup1.cif

Structure factors: contains datablock(s) I. DOI: 10.1107/S1600536811033381/wm2519Isup2.hkl

Additional supplementary materials:  crystallographic information; 3D view; checkCIF report

## Figures and Tables

**Table 1:** Selected bond lengths (Å)

Cd1—N5	2.313 (3)
Cd1—N4	2.363 (3)
Cd1—N1^i^	2.372 (3)
Cd1—N3	2.406 (3)
Cd1—N6	2.443 (4)
Cd1—N2^i^	2.444 (3)

Symmetry code: (i) 
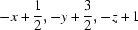
.

**Table 2:** Hydrogen-bond geometry (Å, °)

*D*—H⋯*A*	*D*—H	H⋯*A*	*D*⋯*A*	*D*—H⋯*A*
N1—H1*D*⋯F4^ii^	0.90	2.18	3.083 (4)	176
N2—H2*C*⋯F6^iii^	0.91	2.25	3.076 (4)	150
N3—H3*C*⋯F1^i^	0.91	2.17	3.026 (4)	155
N4—H4*C*⋯F2^iv^	0.90	2.26	3.130 (4)	162
N5—H5*C*⋯F1	0.90	2.11	2.998 (4)	170
N5—H5*D*⋯F5^v^	0.90	2.14	3.013 (4)	165
N6—H6⋯F6^vi^	0.93	2.23	3.134 (5)	164

Symmetry codes: (i) 
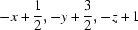
; (ii) 
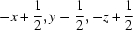
; (iii) 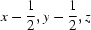; (iv) 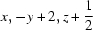; (v) 
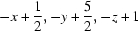
; (vi) 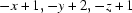.

**Fig. 1.**
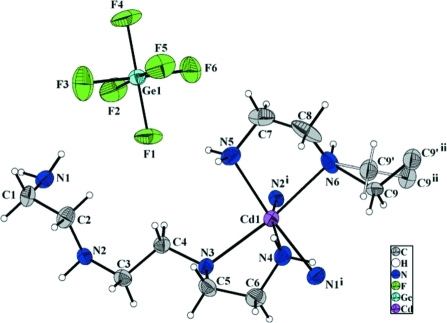
The molecular structure of (I), with the atom-numbering scheme. Displacement ellipsoids are drawn at the 50% probability level. [Symmetry codes: (i) 1/2 - x, 1.5 - y, 1 - z;, (ii) 1 - x, y, 1.5 - z.]

**Fig. 2.**
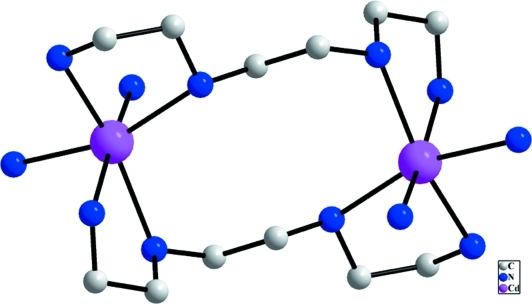
Dimeric Cd2 fragment found in (I); H atoms omitted for clarity.

**Fig. 3.**
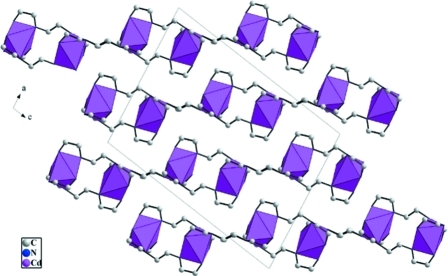
One-dimensional infinite cationic [Cd2(TETA)3]n chains in (I).

**Fig. 4.**
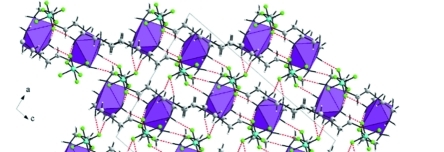
The crystal packing of (I), projected along the b axis.

## supplementary crystallographic information

### Comment

Much interest has been focused on the rational design and construction of microporous materials with intriguing topological architectures and promising applications (Cheetham *et al.*, 1999; Zheng *et al.*, 2003; Zou *et al.*, 2005; Liang *et al.*, 2006; Su *et al.*, 2009). Compared to the rapid development of silicate and phosphate open-framework structures, the progress in the field of fluorides remains moderate. With the exception of some fluoridosilicates, fluoridoaluminates and fluoridotitanates, only a few fluoridogermanates (Brauer *et al.*, 1980, 1986; Lukevics *et al.*, 1997; Wang *et al.*, 2004; Zhang *et al.*, 2003) have been reported. In this structure family, the central Ge(IV) ions are octahedrally coordinated by fluorine atoms. In addition, various organic amines or metal-complex cations have frequently been employed in the synthesis of such fluorides. The main purpose of our work is to explore the construction of novel microporous germanates with Cd^II^ complexes as counter anions. Unexpectedly, the title compound, (I), was obtained, which represents a new fluoridogermanate with one-dimensional Cd^II^ complex cations.

As shown in Fig. 1, the asymmetric unit of (I) contains one unique germanium(IV) atom, one cadmium(II) atom, six fluoride anions, as well as one and a half TETA molecules (TETE = triethylenetetramine, C_6_H_18_N_4_), the latter being completed by a twofold rotation axis. The cadmium atom is six-coordinated in a distorted octahedral geometry by six amine N atoms from three TETA ligands. The Cd—N bond lengths span the range 2.313 (3)–2.444 (3) Å, which are comparable with those observed in other related compounds, e.g. (Bartoszak-Adamska *et al.*, 2002; Ma *et al.*, 2005; Bose *et al.*, 2006). The Ge—F bond lengths in the fluoridogermanate anion are in the range 1.754 (3)–1.788 (2) Å (average 1.772 Å), similar to the distances observed in the inorganic complex K_2_GeF_6_ (Ge—F 1.77 Å) (Hoard & Vincent, 1939), or in other fluoridogermanates, such as [(CH_3_)_4_N][(CF_3_)_3_GeF_2_] (Brauer *et al.*, 1986), [Ni(C_2_H_8_N_2_)(C_6_H_18_N_4_)][GeF_6_] (Wang *et al.*, 2004) and [Ni(dien)_2_][GeF_6_] (Zhang *et al.*, 2003).

The crystal structure of (I) features an undulated cationic chain [Cd_2_(TETA)_3_]^4+^, with discrete [GeF_6_]^2-^ groups serving as the counter anions and occupying the inter-chain spaces. As shown in Fig. 2, two neighboring cadmium atoms are linked by two bridging TETA ligands to form a dimeric Cd_2_ fragment, with Cd···Cd contacts of 6.311 (2) Å. Such dimeric Cd_2_ motifs, which can be considered as secondary building units (SBUs), are interconnected by the third TETA linker to generate one-dimensional corrugated chains parallel the [101] directions (Fig. 3). Adjacent uniform chains are further interlinked and packed together through the [GeF_6_]^2-^ groups into a three-dimensional supramolecular framework (Fig. 4). There are extensive N—H···F hydrogen-bonding interactions between the [GeF_6_]^2-^ ions and amine groups within each of the chains (Table 2).

### Experimental

The title compound was synthesized under hydrothermal conditions. Typically, a mixture of GeO_2_ (0.104 g,1 mmol) CdCO_3_ (0.174 g, 1 mmol), TETA (3.00 ml), pyridine (2.50 ml), hydrofluoric acid (40%, 0.20 ml), and H_2_O (1.00 ml), in a molar ratio 1:1:20:31:10:56, was sealed in a 25 ml Teflon-lined steel autoclave and heated under autogenous pressure at 443 K for 7 days. The block-shaped crystals obtained were recovered by filtration, washed with distilled water and dried in air.

### Refinement

Atom C9 of one TETA is equally disordered about a twofold rotation axis. The H atom bound to atom N6 was located in a difference Fourier map and refined as riding in its as-found position, with N—H = 0.93 Å and *U*_iso_(H) = 1.2*U*_eq_(N). The remaining H atoms were all positioned geometrically and allowd to ride on their parent atoms with C—H = 0.97 Å, N—H = 0.90 (NH_2_) or 0.91 (NH) Å, and with *U*_iso_(H) = 1.2*U*_eq_(C,*N*).

### Crystal data

**Table:**

[Cd_2_(C_6_H_18_N_4_)_3_][GeF_6_]	*F*(000) = 2056
*M**_r_* = 1036.71	*D*_x_ = 2.099 Mg m^−^^3^
Monoclinic, *C*2/*c*	Mo *K*α radiation, λ = 0.71073 Å
Hall symbol: -C 2yc	Cell parameters from 3385 reflections
*a* = 16.5034 (3) Å	θ = 1.9–26.5°
*b* = 9.1072 (3) Å	µ = 3.20 mm^−^^1^
*c* = 22.1920 (4) Å	*T* = 295 K
β = 100.354 (5)°	Block, colorless
*V* = 3281.14 (14) Å^3^	0.10 × 0.06 × 0.05 mm
*Z* = 4	

### Data collection

**Table:**

Bruker APEX area-detector diffractometer	3385 independent reflections
Radiation source: fine-focus sealed tube	2642 reflections with *I* > 2σ(*I*)
graphite	*R*_int_ = 0.045
φ and ω scans	θ_max_ = 26.5°, θ_min_ = 1.9°
Absorption correction: multi-scan (*SADABS*; Sheldrick, 1996)	*h* = −20→20
*T*_min_ = 0.741, *T*_max_ = 0.857	*k* = −11→11
11087 measured reflections	*l* = −27→27

### Refinement

**Table:**

Refinement on *F*^2^	Primary atom site location: structure-invariant direct methods
Least-squares matrix: full	Secondary atom site location: difference Fourier map
*R*[*F*^2^ > 2σ(*F*^2^)] = 0.035	Hydrogen site location: inferred from neighbouring sites
*wR*(*F*^2^) = 0.076	H-atom parameters constrained
*S* = 1.00	*w* = 1/[σ^2^(*F*_o_^2^) + (0.0321*P*)^2^] where *P* = (*F*_o_^2^ + 2*F*_c_^2^)/3
3385 reflections	(Δ/σ)_max_ = 0.001
218 parameters	Δρ_max_ = 0.69 e Å^−^^3^
0 restraints	Δρ_min_ = −0.55 e Å^−^^3^

### Special details

**Table:**

Geometry. All e.s.d.'s (except the e.s.d. in the dihedral angle between two l.s. planes) are estimated using the full covariance matrix. The cell e.s.d.'s are taken into account individually in the estimation of e.s.d.'s in distances, angles and torsion angles; correlations between e.s.d.'s in cell parameters are only used when they are defined by crystal symmetry. An approximate (isotropic) treatment of cell e.s.d.'s is used for estimating e.s.d.'s involving l.s. planes.
Refinement. Refinement of *F*^2^ against ALL reflections. The weighted *R*-factor *wR* and goodness of fit *S* are based on *F*^2^, conventional *R*-factors *R* are based on *F*, with *F* set to zero for negative *F*^2^. The threshold expression of *F*^2^ > σ(*F*^2^) is used only for calculating *R*-factors(gt) *etc*. and is not relevant to the choice of reflections for refinement. *R*-factors based on *F*^2^ are statistically about twice as large as those based on *F*, and *R*- factors based on ALL data will be even larger.

### Fractional atomic coordinates and isotropic or equivalent isotropic displacement parameters (Å^2^)

**Table:**

	*x*	*y*	*z*	*U*_iso_*/*U*_eq_	Occ. (<1)
Ge1	0.33579 (3)	1.10395 (5)	0.374972 (19)	0.02492 (12)	
Cd1	0.329148 (17)	0.90058 (3)	0.624812 (13)	0.02438 (10)	
F1	0.28347 (16)	0.9992 (3)	0.42434 (12)	0.0490 (7)	
F2	0.33638 (19)	0.9446 (3)	0.32858 (12)	0.0551 (8)	
F3	0.24171 (19)	1.1545 (4)	0.32908 (16)	0.0789 (10)	
F4	0.38899 (19)	1.2061 (3)	0.32551 (13)	0.0611 (8)	
F5	0.33215 (19)	1.2603 (3)	0.42160 (13)	0.0561 (8)	
F6	0.43124 (16)	1.0523 (3)	0.41810 (14)	0.0574 (8)	
C1	0.0710 (3)	0.8812 (5)	0.32142 (18)	0.0321 (10)	
H1A	0.0226	0.8239	0.3048	0.039*	
H1B	0.0662	0.9762	0.3013	0.039*	
C2	0.0756 (3)	0.9018 (4)	0.38958 (19)	0.0329 (10)	
H2A	0.1242	0.9586	0.4062	0.039*	
H2B	0.0276	0.9557	0.3969	0.039*	
C3	0.0854 (2)	0.7744 (4)	0.48790 (18)	0.0286 (10)	
H3A	0.0860	0.6776	0.5062	0.034*	
H3B	0.0373	0.8263	0.4964	0.034*	
C4	0.1630 (2)	0.8575 (4)	0.51675 (17)	0.0264 (9)	
H4A	0.2072	0.8312	0.4953	0.032*	
H4B	0.1530	0.9620	0.5110	0.032*	
C5	0.1347 (2)	0.8992 (5)	0.61866 (19)	0.0319 (10)	
H5A	0.0843	0.8424	0.6147	0.038*	
H5B	0.1205	0.9964	0.6023	0.038*	
C6	0.1729 (3)	0.9119 (5)	0.68592 (19)	0.0344 (10)	
H6A	0.1342	0.9585	0.7080	0.041*	
H6B	0.1851	0.8147	0.7030	0.041*	
C7	0.3999 (3)	1.1777 (5)	0.5701 (2)	0.0465 (13)	
H7A	0.3915	1.2742	0.5514	0.056*	
H7B	0.4429	1.1286	0.5532	0.056*	
C8	0.4258 (3)	1.1934 (6)	0.6381 (3)	0.0561 (16)	
H8A	0.4726	1.2593	0.6467	0.067*	
H8B	0.3811	1.2364	0.6551	0.067*	
C9	0.4699 (5)	1.0170 (11)	0.7371 (4)	0.030 (2)	0.50
H9A	0.4210	1.0291	0.7552	0.036*	0.50
H9B	0.4871	0.9153	0.7423	0.036*	0.50
C9'	0.4641 (6)	1.1120 (10)	0.7305 (4)	0.036 (2)	0.50
H9'1	0.4820	1.2135	0.7309	0.043*	0.50
H9'2	0.4152	1.1060	0.7490	0.043*	0.50
N1	0.1450 (2)	0.8060 (4)	0.30945 (15)	0.0321 (8)	
H1C	0.1884	0.8675	0.3163	0.038*	
H1D	0.1376	0.7768	0.2701	0.038*	
N2	0.0791 (2)	0.7592 (3)	0.42059 (14)	0.0262 (8)	
H2C	0.0282	0.7201	0.4078	0.031*	
N3	0.19025 (18)	0.8288 (3)	0.58250 (14)	0.0228 (7)	
H3C	0.1867	0.7302	0.5881	0.027*	
N4	0.2490 (2)	0.9988 (4)	0.69334 (15)	0.0309 (8)	
H4C	0.2765	0.9927	0.7321	0.037*	
H4D	0.2373	1.0938	0.6846	0.037*	
N5	0.3231 (2)	1.0924 (4)	0.55588 (16)	0.0339 (9)	
H5C	0.3168	1.0571	0.5175	0.041*	
H5D	0.2798	1.1505	0.5585	0.041*	
N6	0.4478 (2)	1.0527 (5)	0.66701 (16)	0.0434 (11)	
H6	0.4918	1.0265	0.6480	0.052*	

### Atomic displacement parameters (Å^2^)

**Table:**

	*U*^11^	*U*^22^	*U*^33^	*U*^12^	*U*^13^	*U*^23^
Ge1	0.0254 (2)	0.0250 (2)	0.0255 (2)	0.00042 (19)	0.00756 (19)	0.00145 (19)
Cd1	0.02349 (17)	0.02780 (17)	0.02159 (16)	−0.00060 (14)	0.00335 (12)	0.00203 (13)
F1	0.0584 (18)	0.0460 (16)	0.0511 (17)	−0.0165 (14)	0.0329 (15)	0.0000 (13)
F2	0.089 (2)	0.0400 (16)	0.0407 (17)	−0.0092 (15)	0.0232 (16)	−0.0141 (13)
F3	0.051 (2)	0.096 (3)	0.078 (2)	0.0250 (18)	−0.0195 (17)	0.013 (2)
F4	0.088 (2)	0.0541 (18)	0.0510 (18)	−0.0172 (17)	0.0385 (17)	0.0112 (15)
F5	0.086 (2)	0.0315 (15)	0.0573 (19)	−0.0009 (15)	0.0303 (17)	−0.0134 (13)
F6	0.0343 (16)	0.067 (2)	0.066 (2)	0.0066 (14)	−0.0047 (14)	0.0115 (16)
C1	0.031 (2)	0.037 (3)	0.025 (2)	0.006 (2)	−0.0024 (19)	0.0038 (19)
C2	0.035 (2)	0.033 (2)	0.030 (2)	0.012 (2)	0.004 (2)	0.004 (2)
C3	0.023 (2)	0.034 (2)	0.030 (2)	−0.0027 (19)	0.0072 (19)	−0.0004 (19)
C4	0.024 (2)	0.032 (2)	0.024 (2)	0.0006 (18)	0.0051 (18)	0.0019 (17)
C5	0.023 (2)	0.041 (2)	0.032 (2)	−0.005 (2)	0.0067 (18)	−0.005 (2)
C6	0.038 (3)	0.043 (3)	0.026 (2)	−0.005 (2)	0.014 (2)	−0.006 (2)
C7	0.047 (3)	0.035 (3)	0.061 (4)	−0.005 (2)	0.020 (3)	0.009 (2)
C8	0.041 (3)	0.048 (3)	0.086 (4)	−0.026 (3)	0.029 (3)	−0.036 (3)
C9	0.029 (5)	0.037 (5)	0.024 (5)	−0.012 (4)	0.007 (4)	0.003 (4)
C9'	0.038 (5)	0.037 (5)	0.030 (5)	0.000 (5)	0.001 (4)	0.000 (5)
N1	0.038 (2)	0.0334 (19)	0.0252 (19)	−0.0037 (18)	0.0061 (16)	0.0030 (16)
N2	0.0225 (18)	0.0313 (19)	0.0237 (18)	−0.0041 (15)	0.0014 (15)	−0.0010 (15)
N3	0.0265 (18)	0.0226 (17)	0.0191 (17)	−0.0001 (15)	0.0031 (14)	−0.0004 (14)
N4	0.037 (2)	0.033 (2)	0.0221 (19)	−0.0009 (17)	0.0039 (16)	−0.0029 (15)
N5	0.033 (2)	0.035 (2)	0.035 (2)	0.0049 (18)	0.0080 (17)	0.0095 (17)
N6	0.029 (2)	0.079 (3)	0.023 (2)	−0.017 (2)	0.0078 (17)	−0.015 (2)

### Geometric parameters (Å, °)

**Table:**

Ge1—F6	1.754 (3)	C6—N4	1.468 (5)
Ge1—F3	1.758 (3)	C6—H6A	0.9700
Ge1—F5	1.768 (2)	C6—H6B	0.9700
Ge1—F2	1.780 (2)	C7—N5	1.471 (5)
Ge1—F4	1.786 (2)	C7—C8	1.500 (7)
Ge1—F1	1.788 (2)	C7—H7A	0.9700
Cd1—N5	2.313 (3)	C7—H7B	0.9700
Cd1—N4	2.363 (3)	C8—N6	1.451 (7)
Cd1—N1^i^	2.372 (3)	C8—H8A	0.9700
Cd1—N3	2.406 (3)	C8—H8B	0.9700
Cd1—N6	2.443 (4)	C9—C9'^ii^	1.474 (10)
Cd1—N2^i^	2.444 (3)	C9—N6	1.566 (9)
C1—N1	1.465 (5)	C9—H9A	0.9700
C1—C2	1.513 (6)	C9—H9B	0.9700
C1—H1A	0.9700	C9'—C9^ii^	1.474 (10)
C1—H1B	0.9700	C9'—N6	1.487 (9)
C2—N2	1.466 (5)	C9'—H9'1	0.9700
C2—H2A	0.9700	C9'—H9'2	0.9700
C2—H2B	0.9700	N1—Cd1^i^	2.372 (3)
C3—N2	1.485 (5)	N1—H1C	0.9000
C3—C4	1.526 (5)	N1—H1D	0.9000
C3—H3A	0.9700	N2—Cd1^i^	2.444 (3)
C3—H3B	0.9700	N2—H2C	0.9100
C4—N3	1.471 (5)	N3—H3C	0.9100
C4—H4A	0.9700	N4—H4C	0.9000
C4—H4B	0.9700	N4—H4D	0.9000
C5—N3	1.469 (5)	N5—H5C	0.9000
C5—C6	1.517 (6)	N5—H5D	0.9000
C5—H5A	0.9700	N6—H6	0.9335
C5—H5B	0.9700		
			
F6—Ge1—F3	177.73 (16)	H6A—C6—H6B	108.1
F6—Ge1—F5	91.05 (14)	N5—C7—C8	110.1 (4)
F3—Ge1—F5	90.50 (16)	N5—C7—H7A	109.6
F6—Ge1—F2	89.96 (14)	C8—C7—H7A	109.6
F3—Ge1—F2	88.53 (15)	N5—C7—H7B	109.6
F5—Ge1—F2	178.16 (13)	C8—C7—H7B	109.6
F6—Ge1—F4	89.01 (14)	H7A—C7—H7B	108.1
F3—Ge1—F4	89.32 (16)	N6—C8—C7	111.4 (4)
F5—Ge1—F4	90.70 (13)	N6—C8—H8A	109.3
F2—Ge1—F4	90.85 (13)	C7—C8—H8A	109.3
F6—Ge1—F1	90.45 (13)	N6—C8—H8B	109.3
F3—Ge1—F1	91.21 (15)	C7—C8—H8B	109.3
F5—Ge1—F1	90.02 (12)	H8A—C8—H8B	108.0
F2—Ge1—F1	88.44 (12)	C9'^ii^—C9—N6	112.5 (6)
F4—Ge1—F1	179.11 (14)	C9'^ii^—C9—H9A	109.1
N5—Cd1—N4	100.20 (12)	N6—C9—H9A	109.1
N5—Cd1—N1^i^	171.01 (12)	C9'^ii^—C9—H9B	109.1
N4—Cd1—N1^i^	87.93 (12)	N6—C9—H9B	109.1
N5—Cd1—N3	91.29 (12)	H9A—C9—H9B	107.8
N4—Cd1—N3	75.51 (11)	C9^ii^—C9'—N6	104.0 (6)
N1^i^—Cd1—N3	94.47 (11)	C9^ii^—C9'—H9'1	111.0
N5—Cd1—N6	76.19 (13)	N6—C9'—H9'1	111.0
N4—Cd1—N6	92.37 (12)	C9^ii^—C9'—H9'2	111.0
N1^i^—Cd1—N6	99.84 (13)	N6—C9'—H9'2	111.0
N3—Cd1—N6	160.93 (13)	H9'1—C9'—H9'2	109.0
N5—Cd1—N2^i^	97.66 (11)	C1—N1—Cd1^i^	108.8 (2)
N4—Cd1—N2^i^	161.89 (11)	C1—N1—H1C	109.9
N1^i^—Cd1—N2^i^	74.05 (11)	Cd1^i^—N1—H1C	109.9
N3—Cd1—N2^i^	107.14 (10)	C1—N1—H1D	109.9
N6—Cd1—N2^i^	88.99 (12)	Cd1^i^—N1—H1D	109.9
N1—C1—C2	110.2 (3)	H1C—N1—H1D	108.3
N1—C1—H1A	109.6	C2—N2—C3	112.3 (3)
C2—C1—H1A	109.6	C2—N2—Cd1^i^	108.0 (2)
N1—C1—H1B	109.6	C3—N2—Cd1^i^	122.3 (2)
C2—C1—H1B	109.6	C2—N2—H2C	104.1
H1A—C1—H1B	108.1	C3—N2—H2C	104.1
N2—C2—C1	110.5 (3)	Cd1^i^—N2—H2C	104.1
N2—C2—H2A	109.5	C5—N3—C4	110.8 (3)
C1—C2—H2A	109.5	C5—N3—Cd1	108.2 (2)
N2—C2—H2B	109.5	C4—N3—Cd1	116.0 (2)
C1—C2—H2B	109.5	C5—N3—H3C	107.1
H2A—C2—H2B	108.1	C4—N3—H3C	107.1
N2—C3—C4	111.7 (3)	Cd1—N3—H3C	107.1
N2—C3—H3A	109.3	C6—N4—Cd1	106.8 (2)
C4—C3—H3A	109.3	C6—N4—H4C	110.4
N2—C3—H3B	109.3	Cd1—N4—H4C	110.4
C4—C3—H3B	109.3	C6—N4—H4D	110.4
H3A—C3—H3B	107.9	Cd1—N4—H4D	110.4
N3—C4—C3	114.3 (3)	H4C—N4—H4D	108.6
N3—C4—H4A	108.7	C7—N5—Cd1	109.0 (3)
C3—C4—H4A	108.7	C7—N5—H5C	109.9
N3—C4—H4B	108.7	Cd1—N5—H5C	109.9
C3—C4—H4B	108.7	C7—N5—H5D	109.9
H4A—C4—H4B	107.6	Cd1—N5—H5D	109.9
N3—C5—C6	112.4 (3)	H5C—N5—H5D	108.3
N3—C5—H5A	109.1	C8—N6—C9'	95.0 (5)
C6—C5—H5A	109.1	C8—N6—C9	128.2 (5)
N3—C5—H5B	109.1	C8—N6—Cd1	102.2 (3)
C6—C5—H5B	109.1	C9'—N6—Cd1	124.4 (4)
H5A—C5—H5B	107.9	C9—N6—Cd1	106.9 (4)
N4—C6—C5	110.3 (3)	C8—N6—H6	100.3
N4—C6—H6A	109.6	C9'—N6—H6	119.8
C5—C6—H6A	109.6	C9—N6—H6	109.5
N4—C6—H6B	109.6	Cd1—N6—H6	108.5
C5—C6—H6B	109.6		

Symmetry codes: (i) −*x*+1/2, −*y*+3/2, −*z*+1; (ii) −*x*+1, *y*, −*z*+3/2.

### Hydrogen-bond geometry (Å, °)

**Table:**

*D*—H···*A*	*D*—H	H···*A*	*D*···*A*	*D*—H···*A*
N1—H1D···F4^iii^	0.90	2.18	3.083 (4)	176.
N2—H2C···F6^iv^	0.91	2.25	3.076 (4)	150.
N3—H3C···F1^i^	0.91	2.17	3.026 (4)	155.
N4—H4C···F2^v^	0.90	2.26	3.130 (4)	162.
N5—H5C···F1	0.90	2.11	2.998 (4)	170.
N5—H5D···F5^vi^	0.90	2.14	3.013 (4)	165.
N6—H6···F6^vii^	0.93	2.23	3.134 (5)	164.

Symmetry codes: (iii) −*x*+1/2, *y*−1/2, −*z*+1/2; (iv) *x*−1/2, *y*−1/2, *z*; (i) −*x*+1/2, −*y*+3/2, −*z*+1; (v) *x*, −*y*+2, *z*+1/2; (vi) −*x*+1/2, −*y*+5/2, −*z*+1; (vii) −*x*+1, −*y*+2, −*z*+1.
